# Naturally Occurring Canine Melanoma as a Predictive Comparative Oncology Model for Human Mucosal and Other Triple Wild-Type Melanomas

**DOI:** 10.3390/ijms19020394

**Published:** 2018-01-30

**Authors:** Belen Hernandez, Hibret A. Adissu, Bih-Rong Wei, Helen T. Michael, Glenn Merlino, R. Mark Simpson

**Affiliations:** 1 Laboratory of Cancer Biology and Genetics, Center for Cancer Research, National Cancer Institute, Bethesda, MD 20892, USA; belen.hernandez@nih.gov (B.H.); hibret.adissu@nih.gov (H.A.A.); weib@mail.nih.gov (B.-R.W.); helen.michael@nih.gov (H.T.M.); merlinog@dc37a.nci.nih.gov (G.M.); 2 Medical Research Scholars Program, Office of Clinical Research Training and Medical Education, Clinical Center, National Institutes of Health, Bethesda, MD 20892, USA; 3 Leidos Biomedical Research, Inc., Frederick, MD 21704, USA; 4 NIH Comparative Biomedical Scientist Training Program, Center for Cancer Research, National Cancer Institute, Bethesda, MD 20892, USA

**Keywords:** comparative genomics, clinical trial design, precision medicine, dogs, translational research, drug development, immunotherapy, signal transduction, kinase inhibition

## Abstract

Melanoma remains mostly an untreatable fatal disease despite advances in decoding cancer genomics and developing new therapeutic modalities. Progress in patient care would benefit from additional predictive models germane for human disease mechanisms, tumor heterogeneity, and therapeutic responses. Toward this aim, this review documents comparative aspects of human and naturally occurring canine melanomas. Clinical presentation, pathology, therapies, and genetic alterations are highlighted in the context of current basic and translational research in comparative oncology. Somewhat distinct from sun exposure-related human cutaneous melanomas, there is growing evidence that a variety of gene copy number alterations and protein structure/function mutations play roles in canine melanomas, in circumstances more analogous to human mucosal melanomas and to some extent other melanomas with murine sarcoma viral oncogene homolog B (*BRAF*), Neuroblastoma RAS Viral (V-Ras) Oncogene Homolog (*NRAS*), and neurofibromin 1 tumor suppressor *NF1* triple wild-type genotype. Gaps in canine genome annotation, as well as an insufficient number and depth of sequences covered, remain considerable barriers to progress and should be collectively addressed. Preclinical approaches can be designed to include canine clinical trials addressing immune modulation as well as combined-targeted inhibition of Rat Sarcoma Superfamily/Mitogen-activated protein kinase (RAS/MAPK) and/or Phosphatidylinositol-3-Kinase/Protein Kinase B/Mammalian target of rapamycin (PI3K/AKT/mTOR) signal transduction, pathways frequently activated in both human and canine melanomas. Future investment should be aimed towards improving understanding of canine melanoma as a predictive preclinical surrogate for human melanoma and for mutually benefiting these uniquely co-dependent species.

## 1. Introduction

Melanoma is a significant malignancy, with an estimated 87,110 new cases and 9730 deaths in the United States during 2017 [1]. Melanomas arise from a variety of tissues. Cutaneous melanomas occur most frequently in the skin of people with a fair, non-tanning complexion (phototype 1–2). Although much less frequent, melanomas also arise from other tissues including the uvea of the eye (5.2%) and within mucous membranes (1.3%) [2]. Unlike cutaneous melanoma, non-cutaneous subtypes have a similar incidence across all six dermal phototypes, so noncutaneous melanomas represent a higher proportion of melanomas in many parts of the world [3].

Melanoma pathogenesis is not completely understood. Research continues to address the likelihood of biologically distinct subtypes that differ in cell origin characteristics, the existence of unique clinical and histologic subtypes, as well as varying roles for ultraviolet (UV) radiation, presence of predisposing germ line alterations, and diverse mutational processes [4]. Familial and sporadic genetic risk factors exist [5,6]. Some melanomas are associated with sunlight/UV exposure. Large burdens of mutations typically characterize cutaneous melanomas [7], a feature that adds to the complexity of identifying driver mutations. A significant proportion of cutaneous melanomas harbor recurring (hot spot) mutations in *BRAF* (approximately 50%), *RAS* (approximately 20%), and/or NF1 (approximately 25%) genes, and these mutations can be associated with constitutive activation of the MAPK signaling pathway [7,8,9,10,11,12].

There is also a subgroup of cutaneous melanomas characterized by a lack of *BRAF*, N/H/K-*RAS*, or *NF1* mutations, which are referred to as the triple wild-type (TWT) subtype [13]. The TWT genotype is also a feature underlying most noncutaneous melanomas, including mucosal melanomas [5,14]. Human mucosal melanoma (MM) is known to behave more aggressively and have less favorable prognosis than other melanoma subtypes, possibly due in part to absence of symptoms initially and the occult locations they develop in, which impede early diagnosis [15].

Animal models, notably genetically engineered mouse models, have been invaluable in discerning molecular processes and pathology of cancers including melanoma [16]. Melanoma has been modeled in mice (and zebrafish) engineered to carry defined mutations such as BRAF^V600E^ or NRAS^Q61R/K^ (or G12V), or in some cases through inactivation of tumor suppressor genes such as *CDKN2A* or *PTEN* to model cutaneous melanomas [6,17,18]. Models of TWT cutaneous melanoma are less common, but include the hepatocyte growth factor/scatter factor (HGF) transgenic mouse, which represents a unique model for studying cutaneous TWT melanoma [19,20,21]. Such models have been valuable in elucidating mechanisms of malignant transformation, disease progression, and drug resistance in cutaneous melanoma [6,17]. However, mouse models for non-cutaneous melanomas are still lacking and there is a need for additional suitable animal models [14]. Like all models, mouse models have constraints; examples include limited population heterogeneity, tightly controlled environmental living conditions, and the difficulty of obtaining serial tissue samples.

Naturally occurring cancers in dogs, on the other hand, have several unique advantages as models for human diseases. As in humans, spontaneous cancers in pet dogs typically develop in the presence of an intact immune system and are characterized by tumor growth over an extended period. Inter-individual and intra-tumoral heterogeneity, metastasis, cancer recurrence and therapeutic resistance are all canine cancer disease features [22]. Furthermore, pet dogs and humans share similar environments, which can influence tumor development and progression [23,24]. Investigating canine melanoma can provide an additional avenue for insight into the natural biology of disease, particularly for MM, as these are the most common subtype in dogs. Spontaneous tumors in dogs can provide opportunity for surrogate clinical (preclinical) trials since the heterogeneous naturally evolving disease process occurs in a large immune-competent animal. Care of the dog as a model is more amenable to human-parallel clinical management and discovery than are induced-disease animal models.

## 2. Clinical Manifestations of Canine Melanomas

**Figure 1:**
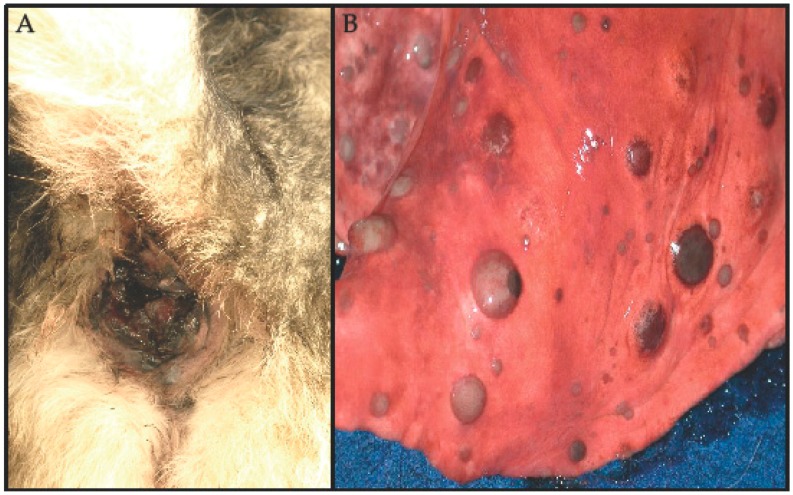
Clinicopathological manifestations of mucosal melanoma in dogs. (**A**) Canine mucosal melanoma involving the anorectal area of a dog. There is a darkly pigmented (melanized) mass involving the mucous membranes of the anus. (**B**) Pulmonary metastasis of a mucosal melanoma. Circumscribed nodular metastatic lesions with varying degrees of melanin pigmentation are disseminated in the lung parenchyma, visible at the visceral pleura, of an autopsy specimen (different dog from image in (**A**)). Lesion photographs were kindly provided by Dr. Jeff Caswell, Department of Pathobiology, Ontario Veterinary College, University of Guelph, Guelph, Ontario, Canada.

Melanoma is a relatively common tumor in dogs [25], with up to 100,000 diagnoses each year in the USA [26,27]. The mean age of dogs with benign and malignant melanocytic neoplasms at diagnosis is 8.1 and 11.6 years, respectively [28]. In dogs, melanocytic malignancies occur most often in the oral cavity (oral/mucosal). Canine melanoma occurs much less frequently in the skin (cutaneous), eye (ocular), the foot pads and nail apparatus (acral), and other mucocutaneous sites (Figure 1). Cutaneous melanocytic neoplasms in dogs generally have an overall favorable prognosis, in contrast to most oral/mucosal and acral melanomas [25,29]. UV is not thought to play a significant role in canine cutaneous melanoma due to the protective hair coat. Anatomic location appears associated with the biological behavior of canine melanocytic neoplasia and therefore is considered a useful prognostic parameter [25].

Canine MM patients have a median survival time ranging from three to 18 months, depending on the stage at diagnosis [25,29,30]. Canine melanomas are associated with breed predispositions and are overrepresented in black-coated dogs [31]. Commonly affected breeds include Scottish terrier, poodle, golden retriever, dachshund, cocker spaniel, and miniature poodle [32]. The higher prevalence in these breeds is thought to reflect, at least in part, genetic predisposition, which may permit identification of inherited (familial) genetic factors and germline mutations in canine melanomas. This could point to related genetic factors in human melanomas [31].

**Figure 2:**
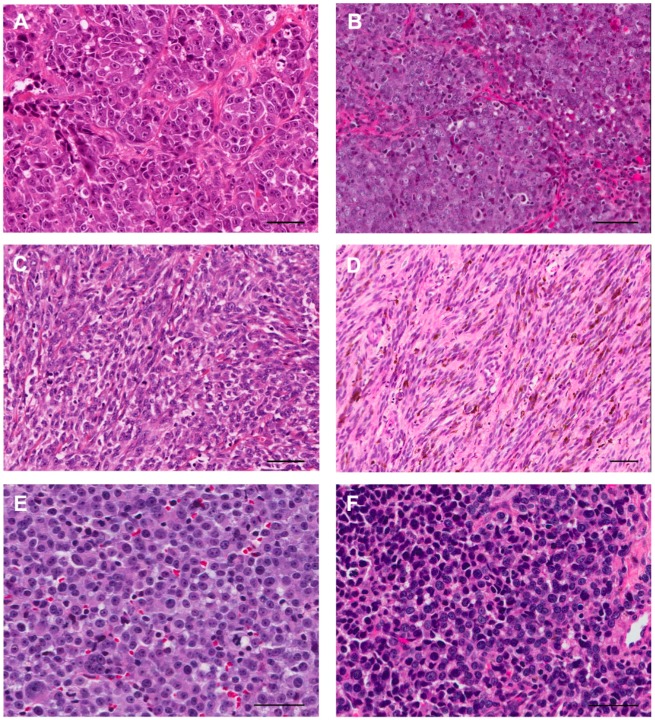
Similarities between histopathological features of human (**A**,**C**,**E**) and canine (**B**,**D**,**F**) mucosal melanomas. Pleomorphic cytomorphologies occurring in both species include (**A**,**B**) epithelioid (polygonal) malignant melanocytes, (**C**,**D**) spindloid malignant melanocytes, and (**E**,**F**) small round blue cell malignant melanocyte morphology. Photomicrographs of hematoxylin and eosin stained tissue sections. Bars = 50 μm. Used by the authors with permission under Creative Commons Attribution License [35].

Canine and human MM share substantial histopathological features (Figure 2) and clinical behavior [31,33,34,35]. According to a consensus study conducted by the National Cancer Institute Comparative Melanoma Tumor Board, a panel of diagnostic and investigative experts with scientific and clinical experience in canine and human melanocytic lesions, these characteristics included melanocyte morphology, patterns of growth including presence of necrosis and ulceration, and the expression of melanocyte differentiation antigens (Table S1) [35]. Canine and human MM share a propensity to metastasize to regional lymph nodes and brain, as well as other visceral organs (Figure 1) [31,33,34,35]. The tumors of both species are generally resistant to chemotherapy and radiation therapy. In veterinary oncology, a standard of care for melanoma is not firmly established. Treatment for dogs with melanoma consists primarily of surgery, with the options of hypofractionated or definitive radiation therapy, and platinum chemotherapy [36,37,38,39].

## 3. Comparative Genetics and Molecular Signaling Pathways

Mainly due to its rarity in humans, relatively little is known about the underlying germline or somatic genetics of the MM subtype compared to cutaneous melanoma. More information about MM is now beginning to emerge [6,40,41,42]. UV exposure is not a risk factor for MM, so tumors lack the high number of UV-signature type mutations found in cutaneous melanoma. Copy number variants appear to more common in human MM than are *BRAF* or *NRAS* mutations, although a few examples with *NRAS* mutations have been documented [5,43] (Table 1).

Similar to human MM, canine MM occur in anatomical locations with limited risk for UV-induced mutations. Although incompletely defined, the genetic/molecular landscape of canine MM appears to more closely resemble human sun-shielded melanomas (mucosal and acral) (Table 1). Analogous *NRAS* mutations do occur infrequently in canine MM [31,44,45,57], but dogs tested thus far generally lack NRAS^G12^ and BRAF^V600^ hotspot mutations (Table 1). While structural rearrangements have not been thoroughly investigated in canine MM, orthologous chromosomal alterations have been identified in dogs with MM [27]. For example, alterations in orthologous chromosome regions encoding MAPK pathway genes occur. *Canis familiaris* chromosome (CFA) 30 harbors MAPK-related genes including Sprouty Related EVH1 Domain Containing 1 (*SPRED1*) and Transient Receptor Potential Cation gene (*TRPM7*), which are involved in suppression and regulation of the MAPK pathway, respectively. The MAPK pathway can be affected by a variety of genomic rearrangements [44,58]. As examples, loss of *SPRED1,* as well as copy number gains or mutation of V-Kit Hardy-Zuckerman 4 Feline Sarcoma Viral Oncogene (*KIT*) and V-Myc Avian Myelocytomatosis Viral Oncogene Homolog (*MYC*) [27], could all contribute to alternative means of promoting MAPK pathway activation in MM. Manifestly, despite the relative infrequency of *BRAF* and *NRAS* mutations, MAPK pathway activation appears to be a feature exhibited fairly commonly in both canine and human MM [40,44,45].

Along with the MAPK pathway, the PI3K/AKT/mTOR pathway can be activated in MM [5,40,59]. Alterations in the PI3K pathway have been shown to arise from inactivating mutation and structural variations. Altered signaling of this nature can be influenced by inactivation of the tumor suppressor *PTEN* [5,60]. In addition to *PTEN*, genes including *TP53* and ubiquitin ligase proto-oncogene (*MDM2*) were found to be mutated in some of the eight human MM studied [5]. Additional efforts to discover precisely if, and how, these molecules may be factors in canine MM are needed.

**Figure 3:**
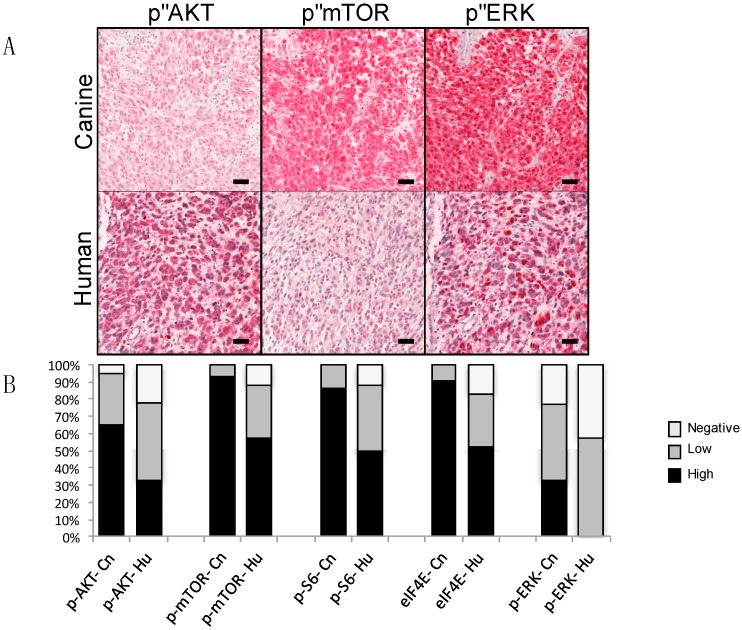
Activation of ERK and PI3K/AKT/mTOR signal transduction pathways in human and canine mucosal melanoma detected by immunohistochemistry on primary tumor tissue arrays. (**A**) Representative immunopositive reactions for pathway mediators are illustrated. Phospho-specific primary antibody signal detection with red chromogen and hematoxylin counterstain. Bars = 50 μm. (**B**) Percentages of immunopositive melanomas for selected canine (Cn, *n* = 43) and human (Hu, *n* = 40) signal transduction mediators. Relative intensity and percentages of immunolabeled cells were considered in scores, assigned as negative, low, and high. Used by the authors with permission under Creative Commons Attribution License [45].

A striking parallel is that both canine [44,45] and human MM [40,45] frequently exhibit RAS/MAPK and/or PI3K/AKT/mTOR signaling pathway activation, which occurs in the absence of some recognized highly recurrent genomic aberration [44,45,61]. An analysis of 40 human and 43 canine primary MM revealed distinct, but variable, p-ERK and/or PI3K/AKT/mTOR activation states in the majority of patients (Figure 3). The precise mechanisms of MAPK and PI3K/AKT/mTOR pathway activation remain largely undetermined in the majority of MM. It is reasonable to assume a number of mechanisms are in play. In some canine MM melanomas, pathway activation may be due loss of *PTEN* [31,50], mutations in *NRAS* occur in a few cases (similar to human MM) [31,45,57], or over-expression of receptor tyrosine kinases, such as platelet derived growth factor receptor (PDGFR) could be possible [56]. Deeper investigations into constitutive activation of critical pathways and the underlying genetic components of canine MM is required to enhance our understanding of biological processes fundamental to canine MM and its utility to model human MM.

In light of the emerging recognition that copy number variations appear to underpin a component of mucosal melanomagenesis in both species, future focus of melanoma genomics should shift towards a wider survey of the genetic landscape. This would contrast with what heretofore has been a narrower focus on single nucleotide variants in limited regions of the genome of MM in particular (Table 1). Analyses shedding additional light in canine MM are anticipated to be forthcoming (unpublished work [62]).

## 4. Canine Mucosal Melanoma as a Preclinical Model

Contemporary study of canine melanoma may serve a dual purpose. In addition to seeking to improve veterinary patient care, more recent therapeutic approaches undertaken in canine MM further efforts toward preclinical development. Prospects include investigating targeted therapies, combinations of drugs and alternate dosing strategies, as well as exploring treatment paradigms such as immunotherapy for treating canine and human melanoma. Canine cell lines and patients with MM provide invaluable resources for in vitro and in vivo investigation of MM and TWT melanoma.

Preclinical development in dogs can mirror human clinical trials [6,24]. In earliest phase, canine clinical trials can reveal critical information about target recognition, drug interactions/toxicity, and also may be designed to provide insight into clinical outcomes of novel treatments and therapeutic agents, which in some cases have been predictive [63,64,65]. Historically, drug safety profile development in the pharmaceutical industry often included dogs [24,35]. Dogs provide an advantage for prospectively planning serial sampling for pharmacokinetic and pharmacodynamic information. Therapeutic development using a canine clinical trial paradigm is only now becoming more organized. Currently, the ability to pilot therapy for humans in dogs lags behind the traditional therapy development pipeline. Consequently, some of the current evidence in the literature regarding therapeutic development in dogs with cancer presented here appears in a somewhat more retrospective light. Despite the present state of development in the dog however, these examples accordingly embody prospective potential for future clinical modeling in the dog with cancer, having relevance for human disease.

### 4.1. Small Molecule Signaling Inhibitors

#### 4.1.1. BRAF, KIT, and MAPK Pathway Inhibitors

Identification of the V600E canonical BRAF mutations in human cutaneous melanoma led to the development of efficacious small molecule inhibitors. Notably, selective BRAF inhibitors, such as Vemurafenib and Dabrafenib, have yielded improved clinical outcomes in melanoma patients compared to conventional chemotherapy [66,67]. Nevertheless, the beneficial effect of these inhibitors as monotherapies is generally short-lived due to acquired drug resistance, and combination therapies of BRAF and other MAPK inhibitors are now used in human patients with improved outcome [68,69].

Comparatively, in vitro studies of canine urinary bladder transitional cell carcinoma harboring orthologous BRAF^V600E^ mutations (e.g., canine V595E) have shown response to the BRAF kinase inhibitor Vemurafenib, while tumor cells with wild-type *BRAF* were unresponsive to the drug [70]. Although canine MM would likely be unresponsive to this BRAF kinase inhibitor, since the ortholog to the V600E mutant is not a frequent event in this tumor type [44,45,48,49,70], the rational targeting of the BRAF mutant is validated in canine cancer.

The existence of activating mutations or gene amplification of the proto-oncogene *KIT* in human MM [11,71] makes KIT a putative therapeutic target. Small molecule kinase inhibitors of KIT, such as Imatinib (Gleevec) and Masitinib, have shown variable success in the treatment of human MM [34,66,72]. Interestingly, clinical response to Imatinib was limited to mucosal melanomas with *KIT* mutations, while tumors with amplification of wild-type *KIT* had no response [73].

In veterinary oncology, feline and canine mast cell tumor and gastrointestinal stromal tumor (GIST) frequently have *KIT* activating mutations and respond to Imatinib [74]. As an example of translational medicine, masitinib mesylate (AB1010) was initially approved in veterinary medicine for the treatment of unresectable canine mast cell tumors activated by *KIT* mutation [75]. Based upon the favorable results achieved in veterinary oncology, masitinib was subsequently investigated for treatment of several human malignancies, such as GIST, mesothelioma, thymoma, thyroid cancer, and colorectal cancer [63]. This presents an elegant example of the manner in which comparative oncology can translate to human clinical therapeutic development. In the case of canine MM, tumor lines were generally unresponsive to KIT inhibitors such as Imatinib, which is consistent with the infrequency of KIT mutation (Table 1) [76].

#### 4.1.2. PI3K Pathway Inhibition and Combined Targeted Therapy

Therapies targeting other pathways, such as PI3K/AKT/mTOR signaling, have induced stable disease in human patients in phase I clinical trials [77]. Similar to the case with BRAF inhibitors, efficacy is limited when used as a single agent [77,78]. PI3K/AKT/mTOR pathway inhibitors have been evaluated in dogs, inhibiting growth of canine melanoma cells in vitro [50,79] and in a canine melanoma xenograft model [45]. Related clinical trials have yet to be conducted.

Growing evidence indicates the majority of human and canine MM tested exhibit RAS/ERK and/or PI3K/AKT/mTOR signaling pathway activation, despite the paucity of canonical *BRAF* and *NRAS* mutations [35,43,44,45]. Inhibition of both pathways may be beneficial due to significant crosstalk and redundancy between the pathways [8,80]. Canine MM cell lines with ERK and AKT/mTOR activation are sensitive to MAPK/Erk kinase (MEK) and PI3K/mTOR inhibitors [44,45]. Through apparent signaling crosstalk analogous to several human cancers [81,82,83], targeting PI3K/mTOR in canine MM, which resulted in diminished downstream p-S6 and eIF4E expression, induced reciprocal activation of p-ERK in some cell lines [45]. Furthermore, targeting both pathways in two-drug combinations (MEK inhibitor trametinib, and combined PI3K and mTOR inhibitor dactolisib) negated the reciprocal ERK phosphorylation in vitro. In addition, such inhibitor combinations synergistically decreased cell survival and solid tumor growth in canine MM xenografts in mice [45]. These findings, as well as those of others [40], provide evidence of synergistic therapeutic efficacy when simultaneously targeting multiple mediators in melanomas with RAS/ERK and/or PI3K/mTOR pathway activation. Given these results, dual inhibition of the MAPK and PI3K pathways may be promising therapeutic targets that warrant clinical evaluation for melanomas with activation of RAS/MAPK and/or PI3K/AKT/mTOR, regardless of specific genomic aberration or constitutive basal level of pathway activation. Opportunities exist to work out various drug combinations and alternative dosing schedules that would require much longer to develop in human patients. Such combined targeted approaches represent a step toward improving current management of canine MM and further establishes naturally occurring melanoma in the dog as a clinical surrogate for developing human melanoma therapeutics [45].

### 4.2. Immunomodulators

Evasion of the immune system is a hallmark of malignancy [84]. Recent advances in cancer immunology have brought forth outstanding breakthroughs positioning immunotherapy at the forefront of cancer treatment in veterinary and human medicine. In the face of limited success of conventional therapies and the short survival associated with mucosal melanoma in both species, canine MM presents an attractive opportunity to dissect mechanisms of immunoevasion and the development and testing of novel immune-based therapeutics. However, deficiencies in our understanding of human and canine comparative immunology may hinder direct translation of test-article immune-based therapeutics between the two species. Despite these differences, immunotherapy presents some attractive strategies against melanoma, including targeting or modulating the immune system through various approaches such as immune checkpoint blockade, adoptively transferred cell therapies, or cancer vaccines. Examples of emerging proof of principle for the potential promise of discovery immunotherapy in canine cancer are highlighted in the following sections.

#### 4.2.1. Immune Checkpoint Blockade

Inhibition of T cell checkpoint molecules such as cytotoxic T-lymphocyte-associated antigen-4 (CTLA-4) and programmed cell dealth-1 (PD-1) using monoclonal antibodies has achieved remarkable success in cancer treatment including melanoma in humans [85,86]. Targeting PD-1 with Lambrolizumab PD-1 monoclonal antibodies produced noteworthy responses against advanced human malignant melanoma [85]. Thus far, there are limited studies characterizing PD-1 immune checkpoints and therapeutics in canine cancers. The canine *PD-1* and *PD-L1* genes are highly conserved [87] and expression of PD-L1 was demonstrated in diverse types of canine tumor cells. This would seem to imply modeling has potential [87,88,89]. In addition, PD-1 was shown to be highly expressed on tumor-infiltrating lymphocytes obtained from canine oral melanoma, suggesting that lymphocytes in canine MM could be functionally exhausted via this mechanism [90]. In another study, canine melanoma tumor cell lines and tumor-infiltrating macrophages upregulated PD-L1 expression upon exposure to interferon-γ, suggesting an important mechanism of tumor-mediated T cell suppression [88]. This provided a backdrop to clinical application of PD-1/PD-L1 inhibitors as novel therapeutic agents for canine cancers [90]. Maekawa and colleagues demonstrated that a canine-chimeric PD-L1 monoclonal antibody enhanced cytokine production and proliferation of dog peripheral blood mononuclear cells [90]. Objective anti-tumor responses were observed in one of seven dogs with oral malignant melanoma and one of two dogs with undifferentiated sarcoma when treated with chimeric anti-PD-L1 at 2 or 5 mg/kg every 2 weeks in a pilot clinical study [90]. The authors propose this as a safe and effective treatment option for canine cancers [90]. Species-appropriate development of antibody-based therapeutics would help pilot optimization of relevant human therapeutic approaches and provide opportunity to mechanistically evaluate immune regulation, tolerance, or other eventual loss of efficacy [35].

#### 4.2.2. Adoptively transferred Cell Therapies

Another breakthrough in cancer immunotherapy involves the ex vivo engineering and targeting of T cells to specific tumor antigens. The recent FDA approval of chimeric antigen receptor (CAR)-T cell therapy for drug-resistant acute lymphoblastic leukemia heralded the arrival of this novel technology into the anticancer arsenal. The general approach to generating tumor specific T cells involves the ex vivo expansion of large numbers of autologous T cells. This is followed by transfection of the cells with tumor specific T cell receptors or by attaching tumor-antigen specific antibodies to the T cells, then re-infusing the modified autologous T cells back into the patient [91]. In the case of CAR technology, T cells are transfected with genes encoding chimeric antibody receptors specific for a tumor antigen, resulting in homing of the T cells to the target cell [92]. It is yet to be determined whether CAR-T cell therapy would benefit melanoma patients. Yet, an earlier version of adaptive transfer of T-lymphocytes achieved some success in treating melanoma [93]. In this earlier work, infiltrating lymphocytes harvested from human melanoma patients and expanded ex vivo demonstrated responses in patients with metastatic melanoma. Subsequently, the applicability of an updated version of this technology was modeled. CAR-T cells targeting tumor-associated antigen gp100 (a melanocyte lineage-specific trans-membrane protein) inhibited melanoma progression in a severe combined immune-deficient (*Prkdc**^scid^***) mouse xenograft model [94].

Immunotargeting of melanoma-associated gp100 has been evaluated in dogs with MM using allogeneic cell vaccines expressing human gp100 [95,96]. The objective responses produced could serve to inform subsequent approaches, including CAR-T cell therapies. Adoptive cell therapy is costly, requires sophisticated techniques available in limited laboratories, and therefore is not routinely applicable to canine melanoma patients [97]. However, the potential for applying adoptive cell-based therapies to dogs with cancer continues to be demonstrated [98]. Investigators determined that HER2-specific canine CAR-T cells, with costimulatory CD28 signaling domains, recognized and killed HER2^+^ canine osteosarcoma cell lines in an antigen-dependent manner. Furthermore, comparison of canine- and human-derived transmembrane CAR, along with signaling domains to reduce the potential immunogenicity of CAR, revealed no functional differences between the two species [98]. Translatability of this study to clinical trials in dogs has yet to be substantiated. However, this study illustrated a successful strategy to generate CAR-expressing canine T cells for future preclinical studies in dogs. Such clinical trials in dogs would help the optimization of T cell therapeutic efficacy and durability, as well as risk assessment pertaining to immune-related toxicities.

#### 4.2.3. Tumor Vaccines

The discovery of immunogenic tumor-associated antigens has promoted the development of various vaccines to induce anti-tumor immune response in canine and human patients [32]. These include but are not limited to whole cell, dendritic cell, and DNA vaccines that have been extensively reviewed elsewhere [32,97]. A recombinant DNA vaccine expressing human tyrosinase, intended for the adjunct treatment of stage II and III canine oral melanoma after loco-regional control [99], induced a cross-reacting humoral response that recognized recombinant human tyrosinase. Oncept™, a United States Department of Agriculture (USDA)-approved cancer vaccine in veterinary medicine, produced meaningful clinical responses and marked prolongation of survival in dogs with metastases in initial studies [100,101,102]. The safety and immunogenicity of tyrosinase DNA vaccines were consequently assessed in a human melanoma trial by the same investigators [103]. Subsequent to the canine studies, vaccine efficacy for immunized dogs with melanoma was retrospectively reviewed. Inconsistencies in vaccine effectiveness for patients were noted [104], and various immunological mechanisms have been proposed as reasons for variable success [97]. Canine immunotherapy clinical trials are informative but, as a model, may not be uniformly predictive for human cancer in all circumstances. However, as this example illustrates, understanding the limitations and mechanisms of canine melanoma vaccines is anticipated to further enhance the development of human DNA vaccines.

#### 4.2.4. Cytokine Therapy

Cytokines represent another therapeutic or adjuvantive approach for melanoma. Anti-inflammatory cytokines and the innate immune system are inherent parts of host response that could be exploited to provoke anti-tumor effects in cancers like mucosal melanoma [97]. Among cytokine therapeutics tested, the most promising results have been observed with interleukin 2 (IL-2). IL-2 is a powerful biological response modifier that has been extensively utilized for modulating anti-tumor immune responses. Treatment of human metastatic melanoma with IL-2 resulted in complete and durable cancer regression, albeit in relatively few patients [105,106]. Due to the durable responses, this cytokine has been licensed for treatment of advanced stage human melanoma in many countries [107]. In dogs with malignant oral melanoma, IL-2, along with granulocyte-macrophage colony-stimulating factor (GM-CSF) secreted by lethally-irradiated transgenic xenogeneic cells, appeared to play a role in significantly increasing survival time when used in combination with intralesional herpes simplex thymidine kinase suicide gene co-administered with ganciclovir [108]. The relatively greater response rate in canine patients could be the result of cytokine supplementation with gene therapy, and similar components of this treatment may be explored for human trials.

## 5. Conclusions and Perspectives

The features of naturally occurring melanoma in dogs make modeling an attractive prospect for both humans and dogs with melanomas. Clinical presentation, pathology, and molecular mechanisms are noteworthy hallmarks shared between humans and dogs with melanoma. The outlook for utilizing host immune response and precision targeted therapy mechanisms of action to predict useful interventional approaches for humans with MM, and other TWT subtypes, appears promising.

Currently, there are gaps in our understanding of the genetic and molecular underpinning of all melanoma subtypes, and the rarity of human MM is a hurdle for more adequate characterization. In order for the dog to help fill these gaps, progress critically depends upon more thorough, deeply penetrating next-generation sequencing, as well as the required validations to better annotate the canine genome (accounting for breed-related polymorphisms). Relatively incomplete annotation of the canine genome, compared to that of humans, continues to hinder comprehensive cataloging of the genetic and molecular landscape of canine MM, and thereby the knowledge essential for comparative genomics. Deficiencies in canine genome annotation and the lack of associated platforms for informatics investigations are current challenges posing significant impediments to advances in canine cancer genetics, and represent areas of critical need for the oncology community. Fortunately, genetic analyses in domestic dogs, including whole genome sequencing, RNA sequencing, and array comparative genomic hybridization (aCGH) can contribute to enhancing canine cancer genomics [109]. These data will provide important comparative insights for extending the current benefits of canine MM as a pre-clinical model for human melanoma.

As melanoma research progresses, ongoing interdisciplinary efforts comparable to the NCI Comparative Melanoma Tumor Board should be sustained [35]. In particular, collaborative clinical trials should advance the annotation of the genetic landscape of the disease in both species, and create trials first in dogs, to inform first-in-human (phase I) studies. A prospective advantage in considering dogs as naturally occurring models for human disease is the existence of infrastructure and expertise for clinical trial design and study execution in dogs that is analogous to human clinical settings. Canine clinical trials can be undertaken to yield insight useful for human cancer therapy, while advancing benefit for cancer understanding and treatment in affected dogs (Table 2). The standard of care for most canine cancers is not well established. This fact presents an opportunity to proceed with clinical trials for novel therapeutics in veterinary medicine without first having to experience both failure of front-line therapy and stepwise trial phases, in harmony with standards of care in veterinary medicine [110,111]. The shorter natural canine lifespan, as well as the relatively rapid disease course of most malignancies, provide for earlier outcome measures compared to human trials [6]. A clinical and scientific strategy recommended by NCI Comparative Melanoma Tumor Board serves as a guideline for canine oncology surrogate clinical trials (Table 2). Careful execution, coupled with the level of sophistication possible for these studies, contributes to better new drug applications. Canine clinical research has begun informing FDA decision-making for human as well as canine therapeutics. As a consequence, comprehension of the value that dog studies hold is growing within the pharmaceutical industry, melanoma research community, and patient advocacy groups. The opportunities, once again, create mutually beneficial prospects from, and for, mankind’s best friend.

## Figures and Tables

**Table 1:** Summary of molecular/genetic findings in canine and human mucosal melanoma.

Molecular/Genetic Evaluation		Canine			Human	
	Specimen	# Affected/# Examined	Finding	Specimen	# Affected/# Examined	Finding
*NRAS*	Tumor	1/12	Silent mutation at codon 52 [44]	PDX	0/17	All lack mutations [42]
	Cell lines	1/5	Q61R mutation [44]	PDX	2/10	1 G12A mutation; 1 G13D mutation [41]
	Tumor	2/28	Q61 mutation [45]	Tumor	8/71	G12, G13, or Q61 mutation [46]
	Cell lines	2/5	Q61 mutation [45]	Tumor; LN	3/8	1 G12C mutation and copy number gain; 1 Q61R mutation; 1 copy number loss [5]
*BRAF*	Tumor	0/12	All lack mutations [44]	PDX	0/10	All lack mutations [41]
	Cell lines	0/5	All lack mutations [44]	PDX	0/17	All lack mutations [42]
	Tumor	0/28	All lack mutations [45]	Tumor	0/19	All lack mutations [47]
	Cell lines	0/5	All lack mutations [45]	Tumor	2/8	1 K486E mutation; 1 copy number gain [5]
	Tumor	0/11	All lack mutations [48]	Tumor	6/74	4 V600E mutations; 1 V600K mutation;
	Cell lines	0/6	All lack mutations [48]			1 N188S mutation [46]
	Tumor	2/47	V600E mutation [49]			
*CDKN2A/p16*	Tumor	14/20	Copy number loss [27]	Tumor	3/8	Copy number loss [5]
	Tumor	10/12	Decreased expression by IHC [50]	Tumor	59/59	12 High CDKN2A expression;
	Cell lines	4/6	Decreased expression by IHC [50]			47 Low CDKN2A expression by IHC [51]
p-ERK	TMA	33/43	IHC immunopositive [45]	TMA	21/37	IHC immunopositive [45]
	Tumor	19/28	ERK activation by WB [45]			
	Cell lines	6/6	Basal p-ERK increased by WB [48]			
	Cell lines	4/4	Basal p-ERK increased by WB; cell lines sensitive to MEK inhibitor [44]			
*GNAQ*				Tumor	13/284	Mutation at codon 209 [52]
*GNA11*				Tumor	14/284	Mutation at codon 209 [52]
*KIT*	Tumor	13/20	Copy number gain [27]	PDX	0/17	All lack mutations [42]
	Tumor	30/61	IHC immunopositive [53]	PDX	2/10	Non-synonymous mutations [41]
	Tumor	20/39	All lack mutations; 20 IHC immunopositive [54]	Tumor	2/8	Non-synonymous mutations [5]
	Tumor	33/34	1 missense mutation;	Tumor	4/19	2 Non-synonymous mutations; 2 Deletions;
			5 synonymous mutations at nt1743;			3 of 4 in hotspot domains [47]
			33 IHC immunopositive [55]	Tumor	5/75	Non-synonymous mutations; 1 of 5 mutations were activating [46]
*MYC*	Tumor	16/20	Copy number gain [27]	PDX	1/10	Single mutation [41]
*NF1*				PDX	0/17	All lack mutations [42]
				PDX	1/10	Frameshift mutation [41]
				Tumor	0/19	All lack mutations [47]
				Tumor	1/8	Copy number loss [5]
				Tumor	13/75	Non-synonymous mutations; 9 of 13 mutations were inactivating [46]
*p53*	Tumor	8/12	Decreased by IHC [50]	Tumor	59/59	12 High expression;
	Cell lines	3/6	Decreased by IHC [50]			47 Low expression by IHC [51]
	Tumor	7/20	6 Copy number loss; 1 Copy number gain [27]	Tumor	2/8	Copy number loss [5]
PDGFR	Tumor	18/48	IHC immunopositive [56]			
*PTEN*	Tumor	10/12	Decreased by IHC [50]	Tumor	1/8	Copy number loss [5]
	Cell lines	3/6	Decreased by IHC and WB [50]	PDX	1/10	Frameshift mutation [41]
p-AKT	TMA	41/43	IHC immunopositive [45]	TMA	31/40	IHC immunopositive [45]
	Tumor	12/28	AKT activation by WB [45]			
	Cell lines	3/5	Basal p-AKT increased by WB; cell lines sensitive to rapamycin [44]			

Note that some of the referenced studies entail a larger sample set that includes mucosal and non-mucosal melanomas. The findings summarized here correspond to mucosal melanomas. Numbers of cases are presented as # affected with molecular/genetic feature out of # total examined. (Table adapted from Simpson RM et al., 2014 [35]). Review of human literature findings limited to 2016 and 2017. No entry in table = peer-reviewed literature not obtained. Tumor = primary tumor lesion tissue, can be either frozen or fixed. Cell lines = individual lines represent either primary or metastatic tumors. LN = metastasis to lymph node. PDX = patient-derived xenograft tumor tissue. TMA = tumor tissue microarray. WB = Western blot. IHC = immunohistochemistry.

**Table 2:** Suggested consideration for canine melanoma surrogate-clinical trial development ^1^ [35].

Elements of Strategy	Fundamental Action/Procedure	Constructive Consideration
**Clinical documentation**		
• Patient data	Presentation/history, duration, previous work up, management	Breed and other background information useful to generate data on incidence
• Gross lesion documentation	Extent of disease. Description of specific anatomic location (not just indication of oral cavity); dimensions in mm, two axes; ulceration, evidence of dissemination	Photograph lesion with a ruler if possible
• Biopsy	Inclusion for diagnostic intent/therapeutic intent (excisional, incisional); preservation for correlative molecular analysis	Consideration of lateral extent as well as vertical depth of invasion; Attention paid to quality of sampling, preservation, QA, and utilization
• Pathology review	Development of features of malignancy for initial assessment for trial enrollments: Proliferation, growth pattern, invasion, and dissemination, etc. Continue refining prognostic summation; Inclusion of IHC panel if needed to establish diagnosis	Capture classical features outlined—Adapt how used initially vs. what becomes useful from adjunct molecular data and outcomes [ 25];
**Clinical staging/prognosis and monitoring**		
• Imaging for dissemination	Ultrasound of lymph nodes to detect metastasis (includes submandibular)	+/− consideration of removal for staging; alternative consideration ultrasound-guided fine needle aspirate cytology for staging
• CT (MRI) imaging evaluation • Biopsy	Lung particularly; lymph node; abdomen Monitoring response to therapy, as appropriate	Consideration of monitoring for brain involvement; inclusion of cranial imaging Lymph nodes or other palpable disease is recommended
• Endpoint assessment	Necropsy examination, with collection of tissue for research, and documentation of extent of disease/host response	
**Quality of life measures**	Assessments of fatigue, cardiac function, mucositis, altered mentation, serial assessments of metabolic and hemotologic toxicity, threshold of toxicity vs. response	Harmonized approach for multicenter trials similar to [ 111]
**Client education**	Informed consent; Should also include education on how the initiative is intended to explore benefits for both dogs and humans; Necropsy education	Necropsy education; emphasis on historical shortcomings impediment to progress. Education design beyond pro forma consent for necropsy
**Follow up**	Directly with owner/clients and indirectly with primary care clinician	
**Genomics**	Global discovery genomics, proteomics and informatic methods: develop and apply. Database and clinical monitoring integration.	

^1^ Strategic approach for trial design represents an initial outline to be developed further with medical and veterinary oncologists, pathologists, and basic and clinical melanoma research investigators for use in developing multidisciplinary trials for piloting therapeutics for human melanoma. Research outcomes are anticipated to produce parallel benefits for canine melanoma patients.

## Supplementary Materials

Supplementary materials can be found at http://www.mdpi.com/1422-0067/19/2/394/s1.

## References

[1] Siegel R.L., Miller K.D., Jemal A. (2017). Cancer Statistics, 2017. CA Cancer J. Clin..

[2] Chang A.E., Karnell L.H., Menck H.R. (1998). The National Cancer Data Base report on cutaneous and noncutaneous melanoma: A summary of 84,836 cases from the past decade. The American College of Surgeons Commission on Cancer and the American Cancer Society. Cancer.

[3] Kong Y., Si L., Zhu Y., Xu X., Corless C.L., Flaherty K.T., Li L., Li H., Sheng X., Cui C. (2011). Large-scale analysis of KIT aberrations in Chinese patients with melanoma. Clin. Cancer Res..

[4] Bastian B.C. (2014). The molecular pathology of melanoma: An integrated taxonomy of melanocytic neoplasia. Annu. Rev. Pathol..

[5] Hayward N.K., Wilmott J.S., Waddell N., Johansson P.A., Field M.A., Nones K., Patch A.M., Kakavand H., Alexandrov L.B., Burke H. (2017). Whole-genome landscapes of major melanoma subtypes. Nature.

[6] Van der Weyden L., Patton E.E., Wood G.A., Foote A.K., Brenn T., Arends M.J., Adams D.J. (2016). Cross-species models of human melanoma. J. Pathol..

[7] Hodis E., Watson I.R., Kryukov G.V., Arold S.T., Imielinski M., Theurillat J.-P., Nickerson E., Auclair D., Li L., Place C. (2012). A Landscape of Driver Mutations in Melanoma. Cell.

[8] Davies M.A., Kopetz S. (2013). Overcoming resistance to MAPK pathway inhibitors. J. Natl. Cancer Inst..

[9] Van ‘t Veer L.J., Burgering B.M., Versteeg R., Boot A.J., Ruiter D.J., Osanto S., Schrier P.I., Bos J.L. (1989). N-ras mutations in human cutaneous melanoma from sun-exposed body sites. Mol. Cell. Biol..

[10] Ascierto P.A., Kirkwood J.M., Grob J.J., Simeone E., Grimaldi A.M., Maio M., Palmieri G., Testori A., Marincola F.M., Mozzillo N. (2012). The role of BRAF V600 mutation in melanoma. J. Transl. Med..

[11] Curtin J.A., Busam K., Pinkel D., Bastian B.C. (2006). Somatic activation of KIT in distinct subtypes of melanoma. J. Clin. Oncol..

[12] Johnson D.B., Puzanov I. (2015). Treatment of NRAS-mutant melanoma. Curr. Treat. Options Oncol..

[13] Network C.G.A. (2015). Genomic Classification of Cutaneous Melanoma. Cell.

[14] Wilkins D.K., Nathan P.D. (2009). Therapeutic opportunities in noncutaneous melanoma. Ther. Adv. Med. Oncol..

[15] Del Vecchio M., Di Guardo L., Ascierto P.A., Grimaldi A.M., Sileni V.C., Pigozzo J., Ferraresi V., Nuzzo C., Rinaldi G., Testori A. (2014). Efficacy and safety of ipilimumab 3mg/kg in patients with pretreated, metastatic, mucosal melanoma. Eur. J. Cancer.

[16] Day C.P., Merlino G., Van Dyke T. (2015). Preclinical mouse cancer models: A maze of opportunities and challenges. Cell.

[17] Perez-Guijarro E., Day C.P., Merlino G., Zaidi M.R. (2017). Genetically engineered mouse models of melanoma. Cancer.

[18] Kaufman C.K., Mosimann C., Fan Z.P., Yang S., Thomas A.J., Ablain J., Tan J.L., Fogley R.D., van Rooijen E., Hagedorn E.J. (2016). A zebrafish melanoma model reveals emergence of neural crest identity during melanoma initiation. Science.

[19] Noonan F.P., Recio J.A., Takayama H., Duray P., Anver M.R., Rush W.L., De Fabo E.C., Merlino G. (2001). Neonatal sunburn and melanoma in mice. Nature.

[20] Jarrett S.G., Novak M., Harris N., Merlino G., Slominski A., Kaetzel D.M. (2013). NM23 deficiency promotes metastasis in a UV radiation-induced mouse model of human melanoma. Clin. Exp. Metastasis.

[21] Noonan F.P., Zaidi M.R., Wolnicka-Glubisz A., Anver M.R., Bahn J., Wielgus A., Cadet J., Douki T., Mouret S., Tucker M.A. (2012). Melanoma induction by ultraviolet A but not ultraviolet B radiation requires melanin pigment. Nat. Commun..

[22] Khanna C., Lindblad-Toh K., Vail D., London C., Bergman P., Barber L., Breen M., Kitchell B., McNeil E., Modiano J.F. (2006). The dog as a cancer model. Nat. Biotechnol..

[23] Nishiya A.T., Massoco C.O., Felizzola C.R., Perlmann E., Batschinski K., Tedardi M.V., Garcia J.S., Mendonça P.P., Teixeira T.F., Zaidan Dagli M.L. (2016). Comparative Aspects of Canine Melanoma. Vet. Sci..

[24] Paoloni M., Khanna C. (2008). Translation of new cancer treatments from pet dogs to humans. Nat. Rev. Cancer.

[25] Smedley R.C., Spangler W.L., Esplin D.G., Kitchell B.E., Bergman P.J., Ho H.Y., Bergin I.L., Kiupel M. (2011). Prognostic markers for canine melanocytic neoplasms: A comparative review of the literature and goals for future investigation. Vet. Pathol..

[26] Bosenberg M., Arnheiter H., Kelsh R. (2014). Melanoma in mankind’s best friend. Pigment Cell Melanoma Res..

[27] Poorman K., Borst L., Moroff S., Roy S., Labelle P., Motsinger-Reif A., Breen M. (2015). Comparative cytogenetic characterization of primary canine melanocytic lesions using array CGH and fluorescence in situ hybridization. Chromosome Res..

[28] Bolon B., Calderwood Mays M.B., Hall B.J. (1990). Characteristics of canine melanomas and comparison of histology and DNA ploidy to their biologic behavior. Vet. Pathol..

[29] Goldschmidt M., Hendrick M. (2002). Tumors of the Skin and Soft Tissues.

[30] MacEwen E.G., Patnaik A.K., Harvey H.J., Hayes A.A., Matus R. (1986). Canine oral melanoma: Comparison of surgery versus surgery plus Corynebacterium parvum. Cancer Investig..

[31] Gillard M., Cadieu E., De Brito C., Abadie J., Vergier B., Devauchelle P., Degorce F., Dreano S., Primot A., Dorso L. (2014). Naturally occurring melanomas in dogs as models for non-UV pathways of human melanomas. Pigment Cell Melanoma Res..

[32] Bergman P.J., Kent M.S., Farese J.P., Page R.L., Withrow S.J., Vail D.M. (2013). Melanoma. Withrow and MacEwen’s Small Animal Clinical Oncology.

[33] Bergman P.J. (2007). Canine oral melanoma. Clin. Tech. Small Anim. Pract..

[34] Carvajal R.D., Spencer S.A., Lydiatt W. (2012). Mucosal melanoma: A clinically and biologically unique disease entity. J. Natl. Compr. Cancer Netw..

[35] Simpson R.M., Bastian B.C., Michael H.T., Webster J.D., Prasad M.L., Conway C.M., Prieto V.M., Gary J.M., Goldschmidt M.H., Esplin D.G. (2014). Sporadic naturally occurring melanoma in dogs as a preclinical model for human melanoma. Pigment Cell Melanoma Res..

[36] Bostock D.E. (1979). Prognosis after surgical excision of canine melanomas. Vet. Pathol..

[37] Blackwood L., Dobson J.M. (1996). Radiotherapy of oral malignant melanomas in dogs. J. Am. Vet. Med. Assoc..

[38] Theon A.P., Rodriguez C., Madewell B.R. (1997). Analysis of prognostic factors and patterns of failure in dogs with malignant oral tumors treated with megavoltage irradiation. J. Am. Vet. Med. Assoc..

[39] Rassnick K.M., Ruslander D.M., Cotter S.M., Al-Sarraf R., Bruyette D.S., Gamblin R.M., Meleo K.A., Moore A.S. (2001). Use of carboplatin for treatment of dogs with malignant melanoma: 27 cases (1989–2000). J. Am. Vet. Med. Assoc..

[40] Meier F., Busch S., Lasithiotakis K., Kulms D., Garbe C., Maczey E., Herlyn M., Schittek B. (2007). Combined targeting of MAPK and AKT signalling pathways is a promising strategy for melanoma treatment. Br. J. Dermatol..

[41] Garman B., Anastopoulos I.N., Krepler C., Brafford P., Sproesser K., Jiang Y., Wubbenhorst B., Amaravadi R., Bennett J., Beqiri M. (2017). Genetic and Genomic Characterization of 462 Melanoma Patient-Derived Xenografts, Tumor Biopsies, and Cell Lines. Cell Rep..

[42] Krepler C., Sproesser K., Brafford P., Beqiri M., Garman B., Xiao M., Shannan B., Watters A., Perego M., Zhang G. (2017). A Comprehensive Patient-Derived Xenograft Collection Representing the Heterogeneity of Melanoma. Cell Rep..

[43] Furney S.J., Turajlic S., Stamp G., Nohadani M., Carlisle A., Thomas J.M., Hayes A., Strauss D., Gore M., van den Oord J. (2013). Genome sequencing of mucosal melanomas reveals that they are driven by distinct mechanisms from cutaneous melanoma. J. Pathol..

[44] Fowles J.S., Denton C.L., Gustafson D.L. (2015). Comparative analysis of MAPK and PI3K/AKT pathway activation and inhibition in human and canine melanoma. Vet. Comp. Oncol..

[45] Wei B.R., Michael H.T., Halsey C.H.C., Peer C.J., Adhikari A., Dwyer J.E., Hoover S.B., El Meskini R., Kozlov S., Weaver Ohler Z. (2016). Synergistic targeted inhibition of MEK and dual PI3K/mTOR diminishes viability and inhibits tumor growth of canine melanoma underscoring its utility as a preclinical model for human mucosal melanoma. Pigment Cell Melanoma Res..

[46] Cosgarea I., Ugurel S., Sucker A., Livingstone E., Zimmer L., Ziemer M., Utikal J., Mohr P., Pfeiffer C., Pfohler C. (2017). Targeted next generation sequencing of mucosal melanomas identifies frequent NF1 and RAS mutations. Oncotarget.

[47] Lyu J., Song Z., Chen J., Shepard M.J., Song H., Ren G., Li Z., Guo W., Zhuang Z., Shi Y. (2017). Whole-exome sequencing of oral mucosal melanoma reveals mutational profile and therapeutic targets. J. Pathol..

[48] Shelly S., Chien M.B., Yip B., Kent M.S., Theon A.P., McCallan J.L., London C.A. (2005). Exon 15 BRAF mutations are uncommon in canine oral malignant melanomas. Mamm. Genome.

[49] Mochizuki H., Kennedy K., Shapiro S.G., Breen M. (2015). BRAF Mutations in Canine Cancers. PLoS ONE.

[50] Koenig A., Bianco S.R., Fosmire S., Wojcieszyn J., Modiano J.F. (2002). Expression and significance of p53, rb, p21/waf-1, p16/ink-4a, and PTEN tumor suppressors in canine melanoma. Vet. Pathol..

[51] Chen H., Li Y., Long Y., Tang E., Wang R., Huang K., Xie C., Chen G. (2017). Increased p16 and p53 protein expression predicts poor prognosis in mucosal melanoma. Oncotarget.

[52] Sheng X., Kong Y., Li Y., Zhang Q., Si L., Cui C., Chi Z., Tang B., Mao L., Lian B. (2016). GNAQ and GNA11 mutations occur in 9.5% of mucosal melanoma and are associated with poor prognosis. Eur. J. Cancer.

[53] Newman S.J., Jankovsky J.M., Rohrbach B.W., LeBlanc A.K. (2012). C-kit expression in canine mucosal melanomas. Vet. Pathol..

[54] Murakami A., Mori T., Sakai H., Murakami M., Yanai T., Hoshino Y., Maruo K. (2011). Analysis of KIT expression and KIT exon 11 mutations in canine oral malignant melanomas. Vet. Comp. Oncol..

[55] Chu P.Y., Pan S.L., Liu C.H., Lee J., Yeh L.S., Liao A.T. (2013). KIT gene exon 11 mutations in canine malignant melanoma. Vet. J..

[56] Iussich S., Maniscalco L., Di Sciuva A., Iotti B., Morello E., Martano M., Gattino F., Buracco P., De Maria R. (2017). PDGFRs expression in dogs affected by malignant oral melanomas: Correlation with prognosis. Vet. Comp. Oncol..

[57] Mayr B., Schaffner G., Reifinger M., Zwetkoff S., Prodinger B. (2003). N-ras mutations in canine malignant melanomas. Vet. J..

[58] Kim H.S., Jung M., Kang H.N., Kim H., Park C.W., Kim S.M., Shin S.J., Kim S.H., Kim S.G., Kim E.K. (2017). Oncogenic BRAF fusions in mucosal melanomas activate the MAPK pathway and are sensitive to MEK/PI3K inhibition or MEK/CDK4/6 inhibition. Oncogene.

[59] Tanami H., Imoto I., Hirasawa A., Yuki Y., Sonoda I., Inoue J., Yasui K., Misawa-Furihata A., Kawakami Y., Inazawa J. (2004). Involvement of overexpressed wild-type BRAF in the growth of malignant melanoma cell lines. Oncogene.

[60] Guldberg P., thor Straten P., Birck A., Ahrenkiel V., Kirkin A.F., Zeuthen J. (1997). Disruption of the MMAC1/PTEN gene by deletion or mutation is a frequent event in malignant melanoma. Cancer Res..

[61] Manca A., Lissia A., Capone M., Ascierto P.A., Botti G., Caraco C., Stanganelli I., Colombino M., Sini M., Cossu A. (2015). Activating PIK3CA mutations coexist with BRAF or NRAS mutations in a limited fraction of melanomas. J. Transl. Med..

[62] Hendricks W., Zismann V. (2017). Somatic inactivating PTPRJ mutations and dysregulated pathways identified in canine melanoma by integrated comparative genomic analysis. BioRxiv.

[63] Marech I., Patruno R., Zizzo N., Gadaleta C., Introna M., Zito A.F., Gadaleta C.D., Ranieri G. (2014). Masitinib (AB1010), from canine tumor model to human clinical development: Where we are?. Crit. Rev. Oncol. Hematol..

[64] Paoloni M., Davis S., Lana S., Withrow S., Sangiorgi L., Picci P., Hewitt S., Triche T., Meltzer P., Khanna C. (2009). Canine tumor cross-species genomics uncovers targets linked to osteosarcoma progression. BMC Genom..

[65] Rusk A., Cozzi E., Stebbins M., Vail D., Graham J., Valli V., Henkin J., Sharpee R., Khanna C. (2006). Cooperative activity of cytotoxic chemotherapy with antiangiogenic thrombospondin-I peptides, ABT-526 in pet dogs with relapsed lymphoma. Clin. Cancer Res..

[66] Eggermont A.M., Robert C. (2011). New drugs in melanoma: It’s a whole new world. Eur. J. Cancer.

[67] McArthur G.A., Ribas A. (2013). Targeting oncogenic drivers and the immune system in melanoma. J. Clin. Oncol..

[68] Chapman P.B., Hauschild A., Robert C., Haanen J.B., Ascierto P., Larkin J., Dummer R., Garbe C., Testori A., Maio M. (2011). Improved survival with vemurafenib in melanoma with BRAF V600E mutation. N. Engl. J. Med..

[69] Dossett L.A., Kudchadkar R.R., Zager J.S. (2015). BRAF and MEK inhibition in melanoma. Expert Opin. Drug Saf..

[70] Decker B., Parker H.G., Dhawan D., Kwon E.M., Karlins E., Davis B.W., Ramos-Vara J.A., Bonney P.L., McNiel E.A., Knapp D.W. (2015). Homologous Mutation to Human BRAF V600E Is Common in Naturally Occurring Canine Bladder Cancer–Evidence for a Relevant Model System and Urine-Based Diagnostic Test. Mol. Cancer Res..

[71] Handolias D., Salemi R., Murray W., Tan A., Liu W., Viros A., Dobrovic A., Kelly J., McArthur G.A. (2010). Mutations in KIT occur at low frequency in melanomas arising from anatomical sites associated with chronic and intermittent sun exposure. Pigment Cell Melanoma Res..

[72] Hodi F.S., Friedlander P., Corless C.L., Heinrich M.C., Mac Rae S., Kruse A., Jagannathan J., Van den Abbeele A.D., Velazquez E.F., Demetri G.D. (2008). Major response to imatinib mesylate in KIT-mutated melanoma. J. Clin. Oncol..

[73] Hodi F.S., Corless C.L., Giobbie-Hurder A., Fletcher J.A., Zhu M., Marino-Enriquez A., Friedlander P., Gonzalez R., Weber J.S., Gajewski T.F. (2013). Imatinib for melanomas harboring mutationally activated or amplified KIT arising on mucosal, acral, and chronically sun-damaged skin. J. Clin. Oncol..

[74] Bonkobara M. (2015). Dysregulation of tyrosine kinases and use of imatinib in small animal practice. Vet. J..

[75] Hahn K.A., Ogilvie G., Rusk T., Devauchelle P., Leblanc A., Legendre A., Powers B., Leventhal P.S., Kinet J.P., Palmerini F. (2008). Masitinib is safe and effective for the treatment of canine mast cell tumors. J. Vet. Intern. Med..

[76] Ito K., Kobayashi M., Kuroki S., Sasaki Y., Iwata T., Mori K., Kuroki T., Ozawa Y., Tetsuka M., Nakagawa T. (2013). The proteasome inhibitor bortezomib inhibits the growth of canine malignant melanoma cells in vitro and in vivo. Vet. J..

[77] Cleary J.M., Shapiro G.I. (2010). Development of phosphoinositide-3 kinase pathway inhibitors for advanced cancer. Curr. Oncol. Rep..

[78] Kwong L.N., Boland G.M., Frederick D.T., Helms T.L., Akid A.T., Miller J.P., Jiang S., Cooper Z.A., Song X., Seth S. (2015). Co-clinical assessment identifies patterns of BRAF inhibitor resistance in melanoma. J. Clin. Investig..

[79] Kent M.S., Collins C.J., Ye F. (2009). Activation of the AKT and mammalian target of rapamycin pathways and the inhibitory effects of rapamycin on those pathways in canine malignant melanoma cell lines. Am. J. Vet. Res..

[80] She Q.B., Halilovic E., Ye Q., Zhen W., Shirasawa S., Sasazuki T., Solit D.B., Rosen N. (2010). 4E-BP1 is a key effector of the oncogenic activation of the AKT and ERK signaling pathways that integrates their function in tumors. Cancer Cell.

[81] Bailey S.T., Zhou B., Damrauer J.S., Krishnan B., Wilson H.L., Smith A.M., Li M., Yeh J.J., Kim W.Y. (2014). mTOR inhibition induces compensatory, therapeutically targetable MEK activation in renal cell carcinoma. PLoS ONE.

[82] Carracedo A., Ma L., Teruya-Feldstein J., Rojo F., Salmena L., Alimonti A., Egia A., Sasaki A.T., Thomas G., Kozma S.C. (2008). Inhibition of mTORC1 leads to MAPK pathway activation through a PI3K-dependent feedback loop in human cancer. J. Clin. Investig..

[83] Zhu Y.R., Min H., Fang J.F., Zhou F., Deng X.W., Zhang Y.Q. (2015). Activity of the novel dual phosphatidylinositol 3-kinase/mammalian target of rapamycin inhibitor NVP-BEZ235 against osteosarcoma. Cancer Biol. Ther..

[84] Hanahan D., Weinberg R.A. (2011). Hallmarks of Cancer: The Next Generation. Cell.

[85] Hamid O., Robert C., Daud A., Hodi F.S., Hwu W.J., Kefford R., Wolchok J.D., Hersey P., Joseph R.W., Weber J.S. (2013). Safety and tumor responses with lambrolizumab (anti-PD-1) in melanoma. N. Engl. J. Med..

[86] Snyder A., Makarov V., Merghoub T., Yuan J., Zaretsky J.M., Desrichard A., Walsh L.A., Postow M.A., Wong P., Ho T.S. (2014). Genetic basis for clinical response to CTLA-4 blockade in melanoma. N. Engl. J. Med..

[87] Maekawa N., Konnai S., Ikebuchi R., Okagawa T., Adachi M., Takagi S., Kagawa Y., Nakajima C., Suzuki Y., Murata S. (2014). Expression of PD-L1 on canine tumor cells and enhancement of IFN-gamma production from tumor-infiltrating cells by PD-L1 blockade. PLoS ONE.

[88] Hartley G., Faulhaber E., Caldwell A., Coy J., Kurihara J., Guth A., Regan D., Dow S. (2017). Immune regulation of canine tumour and macrophage PD-L1 expression. Vet. Comp. Oncol..

[89] Shosu K., Sakurai M., Inoue K., Nakagawa T., Sakai H., Morimoto M., Okuda M., Noguchi S., Mizuno T. (2016). Programmed Cell Death Ligand 1 Expression in Canine Cancer. In Vivo.

[90] Maekawa N., Konnai S., Okagawa T., Nishimori A., Ikebuchi R., Izumi Y., Takagi S., Kagawa Y., Nakajima C., Suzuki Y. (2016). Immunohistochemical Analysis of PD-L1 Expression in Canine Malignant Cancers and PD-1 Expression on Lymphocytes in Canine Oral Melanoma. PLoS ONE.

[91] Muller D., Kontermann R.E. (2010). Bispecific antibodies for cancer immunotherapy: Current perspectives. BioDrugs.

[92] Jain M.D., Davila M.L. (2017). Concise Review: Emerging Principles from the Clinical Application of Chimeric Antigen Receptor T Cell Therapies for B Cell Malignancies. Stem Cells.

[93] Rosenberg S.A., Dudley M.E. (2009). Adoptive cell therapy for the treatment of patients with metastatic melanoma. Curr. Opin. Immunol..

[94] Zhang G., Wang L., Cui H., Wang X., Zhang G., Ma J., Han H., He W., Wang W., Zhao Y. (2014). Anti-melanoma activity of T cells redirected with a TCR-like chimeric antigen receptor. Sci. Rep..

[95] Gyorffy S., Rodriguez-Lecompte J.C., Woods J.P., Foley R., Kruth S., Liaw P.C., Gauldie J. (2005). Bone marrow-derived dendritic cell vaccination of dogs with naturally occurring melanoma by using human gp100 antigen. J. Vet. Intern. Med..

[96] Alexander A.N., Huelsmeyer M.K., Mitzey A., Dubielzig R.R., Kurzman I.D., Macewen E.G., Vail D.M. (2006). Development of an allogeneic whole-cell tumor vaccine expressing xenogeneic gp100 and its implementation in a phase II clinical trial in canine patients with malignant melanoma. Cancer Immunol. Archol. Immunother..

[97] Atherton M.J., Morris J.S., McDermott M.R., Lichty B.D. (2016). Cancer immunology and canine malignant melanoma: A comparative review. Vet. Immunol. Immunopathol..

[98] Mata M., Vera J.F., Gerken C., Rooney C.M., Miller T., Pfent C., Wang L.L., Wilson-Robles H.M., Gottschalk S. (2014). Toward immunotherapy with redirected T cells in a large animal model: Ex vivo activation, expansion, and genetic modification of canine T cells. J. Immunother..

[99] Bergman P.J. (2014). Immunotherapy in veterinary oncology. Vet. Clin. N. Am. Small Anim. Pract..

[100] Bergman P.J., McKnight J., Novosad A., Charney S., Farrelly J., Craft D., Wulderk M., Jeffers Y., Sadelain M., Hohenhaus A.E. (2003). Long-term survival of dogs with advanced malignant melanoma after DNA vaccination with xenogeneic human tyrosinase: A phase I trial. Clin. Cancer Res..

[101] Grosenbaugh D.A., Leard A.T., Bergman P.J., Klein M.K., Meleo K., Susaneck S., Hess P.R., Jankowski M.K., Jones P.D., Leibman N.F. (2011). Safety and efficacy of a xenogeneic DNA vaccine encoding for human tyrosinase as adjunctive treatment for oral malignant melanoma in dogs following surgical excision of the primary tumor. Am. J. Vet. Res..

[102] Liao J.C., Gregor P., Wolchok J.D., Orlandi F., Craft D., Leung C., Houghton A.N., Bergman P.J. (2006). Vaccination with human tyrosinase DNA induces antibody responses in dogs with advanced melanoma. Cancer Immunol. Arch..

[103] Wolchok J.D., Srinivasan R., Perales M.A., Houghton A.N., Bowne W.B., Blachere N.E. (2001). Alternative roles for interferon-gamma in the immune response to DNA vaccines encoding related melanosomal antigens. Cancer Immunol. Arch..

[104] Ottnod J.M., Smedley R.C., Walshaw R., Hauptman J.G., Kiupel M., Obradovich J.E. (2013). A retrospective analysis of the efficacy of Oncept vaccine for the adjunct treatment of canine oral malignant melanoma. Vet. Comp. Oncol..

[105] Rosenberg S.A., Yang J.C., Topalian S.L., Schwartzentruber D.J., Weber J.S., Parkinson D.R., Seipp C.A., Einhorn J.H., White D.E. (1994). Treatment of 283 consecutive patients with metastatic melanoma or renal cell cancer using high-dose bolus interleukin 2. JAMA.

[106] Rosenberg S.A., Yang J.C., White D.E., Steinberg S.M. (1998). Durability of complete responses in patients with metastatic cancer treated with high-dose interleukin-2: Identification of the antigens mediating response. Ann. Surg..

[107] Atkins M.B. (2002). Interleukin-2: Clinical applications. Semin. Oncol..

[108] Finocchiaro L.M., Fiszman G.L., Karara A.L., Glikin G.C. (2008). Suicide gene and cytokines combined nonviral gene therapy for spontaneous canine melanoma. Cancer Gene Ther..

[109] Briggs J., Paoloni M., Chen Q.-R., Wen X., Khan J., Khanna C. (2011). A Compendium of Canine Normal Tissue Gene Expression. PLoS ONE.

[110] Gordon I., Paoloni M., Mazcko C., Khanna C. (2009). The Comparative Oncology Trials Consortium: Using Spontaneously Occurring Cancers in Dogs to Inform the Cancer Drug Development Pathway. PLoS Med..

[111] Paoloni M., Vail D. (2013). Clinical Trials and Developmental Therapeutic.

