# Supplementary material for: Naturally Occurring Canine Melanoma as a Predictive Comparative Oncology Model for Human Mucosal and Other Triple Wild-Type Melanomas
Source: Int J Mol Sci. 2018 Jan 30;19(2):394. doi: 10.3390/ijms19020394 (PMC5855616; doi:10.3390/ijms19020394)
Supplement: Supplementary file 1 [file ijms-19-00394-s001.pdf]

Table S1. Mucosal melanoma histopathological features shared between humans and dogs.

Malignant melanoma features evaluated	Humans	Dogs
Range of melanocyte morphology		
Epithelioid (polygonal)	√	√ (27/64)[1]
Spindloid (sarcomatoid)	√	√ (9/64)
Mixed epithelioid/ spindloid	√	√ (18/64)
Small round blue cell (undifferentiated)	√	√ (7/64)
Multinucleated	√	√ (3/64)
In situ (lentiginous) mucosal involvement; intraepithelial clusters of malignant melanocytes; clustered and /or individual melanocytes exhibiting superficial ascension in squamous mucosa	√	√ (21/46)
Proprial-submucosal—extent of involvement (vertical growth)	√	√
Extension to subcutaneous (muscularis) involvement	√	√
Necrosis	√	√
Ulceration	√	√
Features of anaplasia, bizarre mitoses, prominent nucleoli	√	√
Invasion (vascular)	√	√
Invasion (bone/cartilage)	√	√
Metastasis	√	√
Amelanotic melanomas	√	√
Expression of melanocyte differentiation antigens	√	√

Features occurring in human melanomas observed (√) in specimens evaluated.
[1] Relative frequency observed in dogs (# observed / # canine patients reviewed; (not quantified for all features, or for all patients).
Features of human mucosal melanoma not observed in either human or canine mucosal malignant melanoma specimens in this study included neoplastic involvement limited to the mucosa in situ, +/- microinvasion (of submucosa); desmoplasia; and neural tissue invasion.
Used by the authors with permission under Creative Commons Attribution License.
Pigment Cell Melanoma Res. 2014 Jan; 27(1): 37–47.
